# Metabolic syndrome is associated with prognosis in patients with acute ischemic stroke after intravenous thrombolysis: a prospective cohort study

**DOI:** 10.3389/fneur.2025.1598434

**Published:** 2025-07-01

**Authors:** Wenyan Chen, Dandan Liu, Zhiqiang Li, Xiaofang Zhang

**Affiliations:** ^1^Department of Geriatric Medicine, Shanxi Bethune Hospital, Shanxi Academy of Medical Sciences, Third Hospital of Shanxi Medical University, Tongji Shanxi Hospital, Taiyuan, China; ^2^Department of Neurology, Linfen People’s Hospital, Linfen, China; ^3^Department of Neurology, Beijing Yanhua Hospital, Beijing, China

**Keywords:** acute ischemic stroke, intravenous thrombolysis, prognosis, metabolic syndrome, mortality

## Abstract

**Objective:**

Metabolic syndrome (MetS) is a known risk factor for stroke, but its impact on prognosis after intravenous thrombolysis (IVT) in acute ischemic stroke (AIS) remains unclear. This study aimed to evaluate the association between MetS and prognosis in AIS patients treated with IVT.

**Methods:**

We conducted a prospective cohort study including AIS patients who received IVT at Shanxi Bethune Hospital between January 2022 and December 2023. Propensity score matching (PSM) was applied to balance baseline characteristics between MetS and non-MetS groups. The primary outcome was 3-month all-cause mortality. Secondary outcomes included good functional outcome (modified Rankin Scale [mRS] 0–2) and symptomatic intracranial hemorrhage (SICH).

**Results:**

After PSM, 292 patients (146 pairs) were enrolled in the analysis. The all-cause mortality rate within 3 months was significantly higher in the MetS group compared to the non-MetS group (24.0% vs. 11.6%; *p* < 0.01). Multivariate Cox regression analysis indicated that MetS was independently associated with increased 3-month mortality (adjusted hazard ratio [HR] = 2.50, 95% CI: 1.35–4.60; *p* < 0.01). A dose–response relationship was observed between the number of MetS components and mortality. Additionally, patients with MetS were less likely to achieve good functional outcomes (adjusted odds ratio [OR] = 0.47, 95% CI: 0.28–0.77; *p* < 0.01) and had a higher risk of SICH (adjusted OR = 2.40, 95% CI: 1.17–4.92; *p* = 0.02).

**Conclusion:**

MetS is an independent predictor of increased mortality, poorer functional recovery, and higher risk of SICH in AIS patients treated with IVT. Early identification and management of metabolic risk factors may improve outcomes in this population.

## Introduction

Acute ischemic stroke (AIS) is a leading cause of global morbidity and mortality worldwide, accounting for approximately 87% of all stroke cases (1). Despite advancements in reperfusion therapies—such as intravenous thrombolysis (IVT) with tissue plasminogen activator (tPA) and endovascular thrombectomy (EVT)—many stroke survivors experience persistent disability or death, underscoring the importance of identifying modifiable prognostic factors (2).

Metabolic syndrome (MetS), characterized by central obesity, hyperglycemia, dyslipidemia, and hypertension, is a recognized risk factor for cardiovascular disease (CVD) and stroke (3). Epidemiological studies show that MetS increases the risk of CVD by 2-fold and stroke by 1.5-fold (4). In recent years, research interest has extended beyond stroke prevention to its influence on post-stroke recovery. Mechanistically, MetS may impair neurological recovery after AIS by promoting systemic inflammation, endothelial dysfunction, and a prothrombotic state, all of which hinder neurovascular repair and exacerbate ischemic injury (5). However, findings on the relationship between MetS and stroke prognosis remain inconsistent. Some studies suggest that MetS independently predicts poorer outcomes and report a dose–response relationship between the number of MetS components and functional impairment (5). In contrast, other studies argue that the predictive value of MetS may primarily reflect the effect of its individual components rather than the syndrome as a whole (6). These discrepancies may be due to heterogeneity in patient populations, stroke subtypes, and treatment strategies across studies.

Among the currently approved therapies, IVT with tPA remains the cornerstone of acute reperfusion treatment for eligible AIS patients. As IVT-treated patients represent a more homogeneous group in terms of treatment protocol and timing, they provide a suitable population to assess the prognostic implications of MetS in a standardized clinical context. This study aimed to evaluate the association between MetS and prognosis in AIS patients receiving IVT.

## Materials and methods

### Patients

This prospective cohort study was conducted at Shanxi Bethune Hospital, a tertiary medical center, from January 2022 to December 2023. Consecutive patients aged ≥18 years who were admitted with a confirmed diagnosis of AIS by computed tomographic (CT) or magnetic resonance imaging (MRI) within 24 h of symptom onset were enrolled. Patients with a modified Rankin Scale (mRS) score ≥ 3 before stroke were excluded to minimize confounding from pre-existing disability, which could independently affect post-stroke outcomes. This criterion is consistent with prior studies that used similar cutoffs to ensure a more functionally homogeneous cohort (7, 8). Additional exclusion criteria included hemorrhagic transformation at admission, perioperative stroke (i.e., stroke occurring during or within 30 days of major surgery), life-threatening comorbidities (e.g., advanced malignancies), pregnancy, participation in other interventional clinical trials during the study period and incomplete clinical or follow-up data.

The study adhered to the ethical guidelines of the Declaration of Helsinki and was approved by the Ethics Committee of Shanxi Bethune Hospital (No. 20211033). Informed consent was provided by participants or legal representatives.

### Definition of MetS

The MetS is a cluster of metabolic abnormalities and there are several definitions. In this study, we adopted the criteria proposed by the 2020 Chinese Diabetes Society (CDS) (9). The definition of MetS status requires the presence of any three or more of the following criteria: (1) abdominal obesity: waist circumference (WC) ≥ 90 cm in men or ≥85 cm in women; (2) hyperglycemia: fasting blood glucose (FBG) ≥ 6.1 mmol/L or 2-h postprandial glucose ≥7.8 mmol/L or previously diagnosed diabetes with treatment; (3) hypertension: systolic/diastolic blood pressure ≥130/85 mm Hg or currently under antihypertensive therapy; (4) triglycerides (TGs) ≥ 1.70 mmol/L; (5) high-density lipoprotein cholesterol (HDL-C) < 1.04 mmol/L. Patients were categorized into two groups based on the presence or absence of MetS.

### Data collection and outcomes

Clinical data were extracted from electronic medical records, including patient’s demographics (age and sex), MetS-related metrics (WC, systolic blood pressure [SBP], diastolic blood pressure [DBP], fasting glucose, triglyceride, and total cholesterol [TC]), medical history (hypertension, atrial fibrillation, coronary artery disease, and previous stroke or transient ischemic attack), National Institutes of Health Stroke Scale (NIHSS), imaging data (trial of ORG 10,172 in Acute Stroke Treatment [TOAST] classification, target artery occlusion and Alberta Stroke Program Early Computed Tomography Score [ASPECTS]), and procedure-related characteristics (onset-to-treatment time and puncture to recanalization time). The baseline stroke severity was assessed by trained neurologists using the NIHSS. Ischemic stroke subtype was classified based on the TOAST classification. ASPECTS was used to evaluate the extent of preoperative early cerebral ischemia.

The primary outcome of this study was the rate of all-cause mortality within 3 months after enrollment. The secondary outcomes include the good functional outcome defined by a mRS of 0–2 at 3 months and the occurrence of symptomatic intracranial hemorrhage (SICH) within 3 months. Follow-up data were collected through medical records review or telephone interviews conducted by trained study coordinators who were blinded to the exposure status.

### Statistical analysis

Continuous variables were tested for normality using the Shapiro–Wilk test. Normally distributed data were expressed as mean ± standard deviation (SD) and compared using Student’s t-test, while non-normally distributed data were presented as median (interquartile range, IQR) and compared using the Mann–Whitney U test. Categorical variables were expressed as frequencies (%) and compared using the chi-squared test or Fisher’s exact test, as appropriate.

To reduce the potential bias caused by baseline differences between MetS and non-MetS groups, propensity score matching (PSM) was performed. Propensity scores were calculated using a logistic regression model that included patient’s demographics, medical history, NIHSS score, imaging data, and procedure-related characteristics as covariates. Patients in the MetS group were matched 1:1 with those in the non-MetS group based on their propensity scores, using a nearest-neighbor matching algorithm without replacement and a caliper width of 0.2 of the standard deviation of the logit of the propensity score. Balance between the matched groups was assessed using standardized mean differences, with a threshold of <0.1 indicating adequate balance.

Based on an anticipated 12% difference in 3-month all-cause mortality between the MetS and non-MetS groups, we performed a post-hoc power analysis using the final matched sample size of 146 patients per group (total n = 292). Assuming a two-sided *α* level of 0.05, the calculated power exceeded 80%, indicating that the study was adequately powered to detect a statistically significant difference in mortality outcomes between the two groups.

Although PSM reduces baseline differences, we further applied multivariate regression analyses after matching to adjust for potential residual confounding and to enhance the precision of effect estimates, as recommended by prior studies (10). To evaluate the primary outcome, multivariate Cox proportional hazards regression analyses were used to assess the association between MetS and 3-month all-cause mortality, controlling for potential confounders including demographic characteristics, medical history, NIHSS, imaging findings, and procedure-related characteristics. In addition, multivariate logistic regression analyses were applied to evaluate the associations between MetS and secondary outcomes, including favorable functional outcome and SICH.

Statistical significance was defined as two-tailed *p* < 0.05. Statistical analysis was performed using the SPSS 22.0 for Windows (SPSS, IBM, United States).

## Results

**Figure 1:**
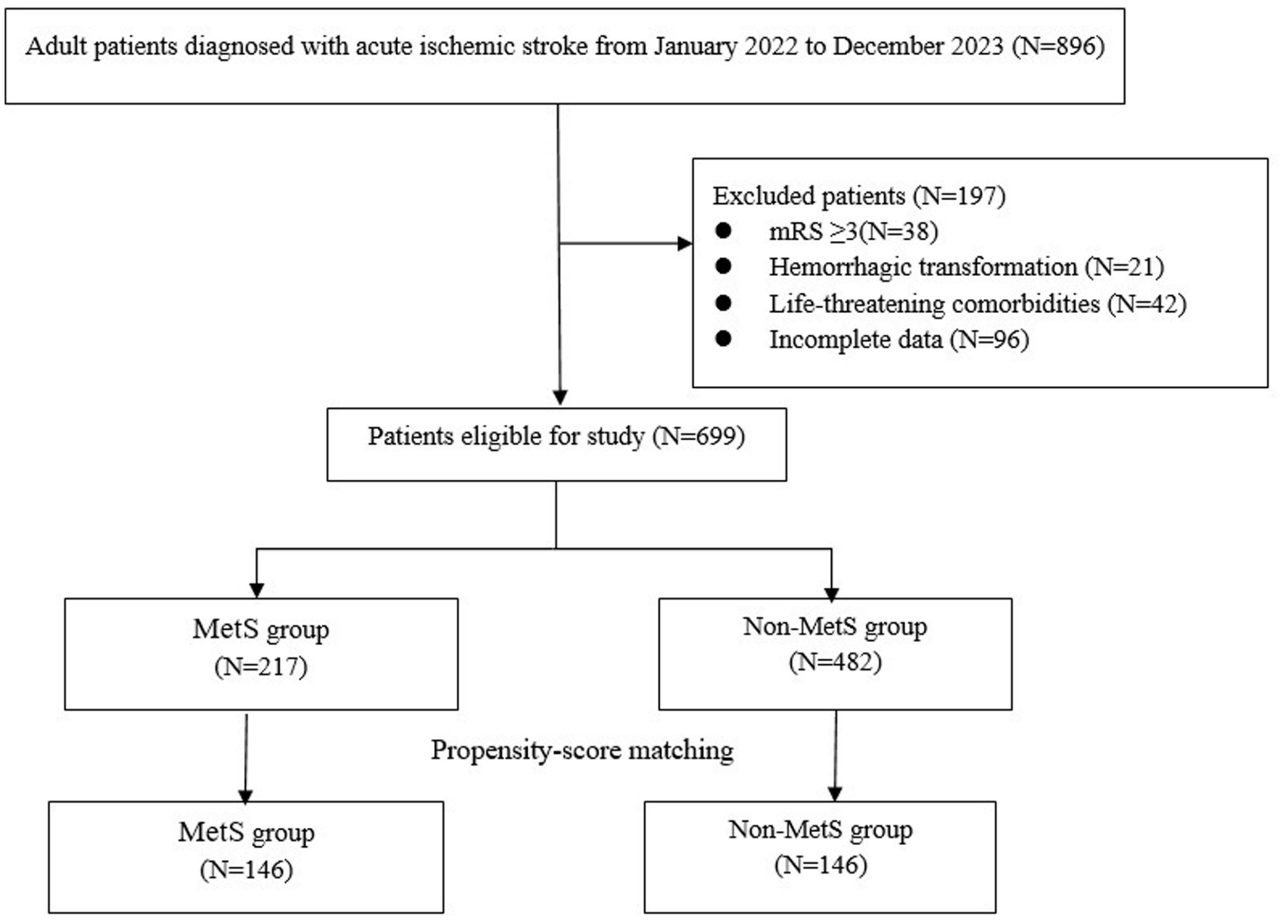
Patient selection flow.

A total of 896 patients with AIS were initially enrolled in this study. After excluding 197 cases, the final analysis included 699 participants, among whom 217 (31.0%) were identified as having MetS. PSM yielded 146 matched pairs, resulting in 292 patients for subsequent comparisons (Figure 1). Baseline characteristics were well-balanced between the two groups, with no significant differences in age, sex, NIHSS score, TOAST classification, medical history, target artery occlusion, or processing times (all *p* > 0.05). However, significant differences were observed in several MetS-related parameters, including SBP, DBP, fasting glucose, triglyceride levels, and TC (all *p* < 0.01), consistent with the diagnostic criteria for MetS (Table 1).

**Table 1:** Baseline characteristics and processing times of patients in the MetS and non-MetS groups.

Characteristics	MetS group	Non-MetS group	*p* value
	(*N* = 146)	(*N* = 146)	
Demographics			
Age, years	68.1 ± 4.4	68.5 ± 4.8	0.40
Male, *n* (%)	82 (56.2%)	77 (52.7%)	0.56
MetS-related metrics			
MetS number, *n* (%)	3.6 ± 0.8	1.8 ± 0.6	<0.01
WC, cm	89.3 ± 8.1	82.2 ± 7.0	<0.01
SBP, mmHg	142.3 ± 13.8	121.2 ± 10.8	<0.01
DBP, mmHg	88.2 ± 12.0	72.1 ± 10.4	<0.01
Fasting glucose, mmol/L	8.4 ± 1.2	5.2 ± 1.0	<0.01
Triglyceride, mmol/L	1.6 ± 0.3	1.1 ± 0.3	<0.01
TC, mmol/L	4.8 ± 0.6	4.2 ± 0.6	<0.01
Medical history, *n* (%)			
Atrial fibrillation	75 (51.4%)	88 (60.3%)	0.13
Coronary artery disease	19 (13.0%)	19 (13.0%)	1.00
Previous stroke or TIA	31 (21.2%)	34 (23.3%)	0.67
NIHSS score	11.3 ± 3.3	11.9 ± 3.5	0.15
TOAST classification, *n* (%)			0.11
LAA	29 (19.9%)	47 (32.2%)	
CE	84 (57.5%)	74 (50.7%)	
OE	2 (1.4%)	2 (1.4%)	
UE	31 (21.2%)	23 (15.8%)	
Target artery occlusion, *n* (%)			
MCA M2	23 (15.8%)	18 (12.3%)	0.40
MCA M1	83 (56.8%)	70 (47.9%)	0.13
Intracranial ICA	31 (21.2%)	38 (26.0%)	0.34
Extracranial ICA	28 (19.2%)	35 (24.0%)	0.32
ASPECTS on the decision image, *n* (%)			0.31
9–10	37 (25.3%)	44 (30.1%)	
7–8	78 (53.4%)	65 (44.5%)	
<7	31 (21.2%)	37 (25.3%)	
Processing times, minutes			
Onset-to-treatment time	228.6 ± 20.8	227.3 ± 22.3	0.59
Puncture to recanalization time	59.1 ± 10.5	57.9 ± 9.4	0.31

ASPECTS, Alberta Stroke Program Early CT Score; CE, cardio embolism; DBP, diastolic blood pressure; ICA, internal carotid artery; LAA, large artery atherosclerosis; MCA, middle cerebral artery; NIHSS, National Institutes of Health Stroke Scale; OE, other etiology; SBP, systolic blood pressure; TC, total cholesterol; TIA, transient ischemic attack; TOAST, Trial of ORG 10172 in Acute Stroke Treatment; MetS, metabolic syndrome; UE, undetermined etiology; WC, waist circumference.

### Primary outcome: all-cause mortality

**Figure 2:**
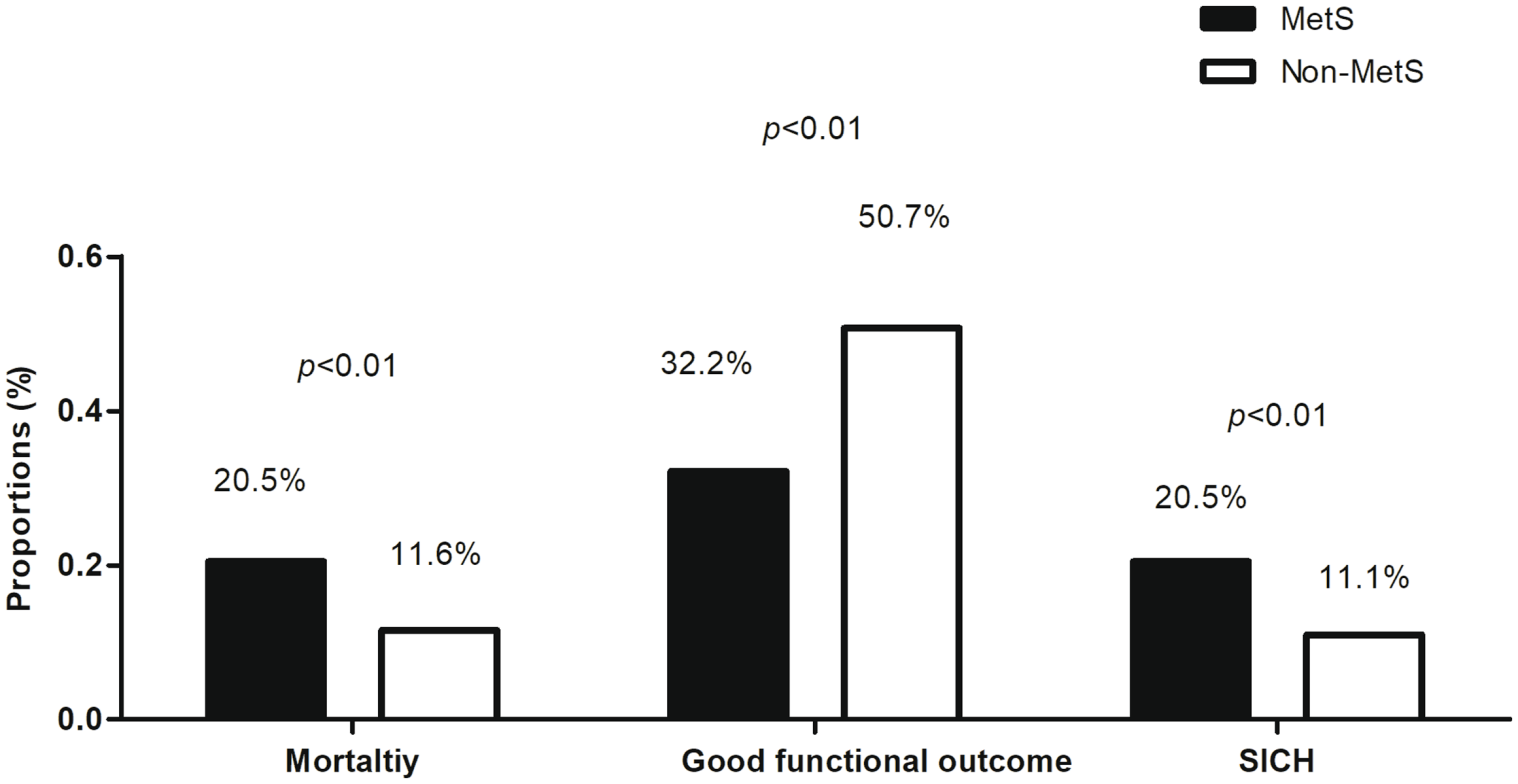
Comparison of the proportions of all-cause mortality, favorable functional outcomes, and symptomatic intracranial hemorrhage (SICH) between patients with and without metabolic syndrome (MetS).

**Figure 3:**
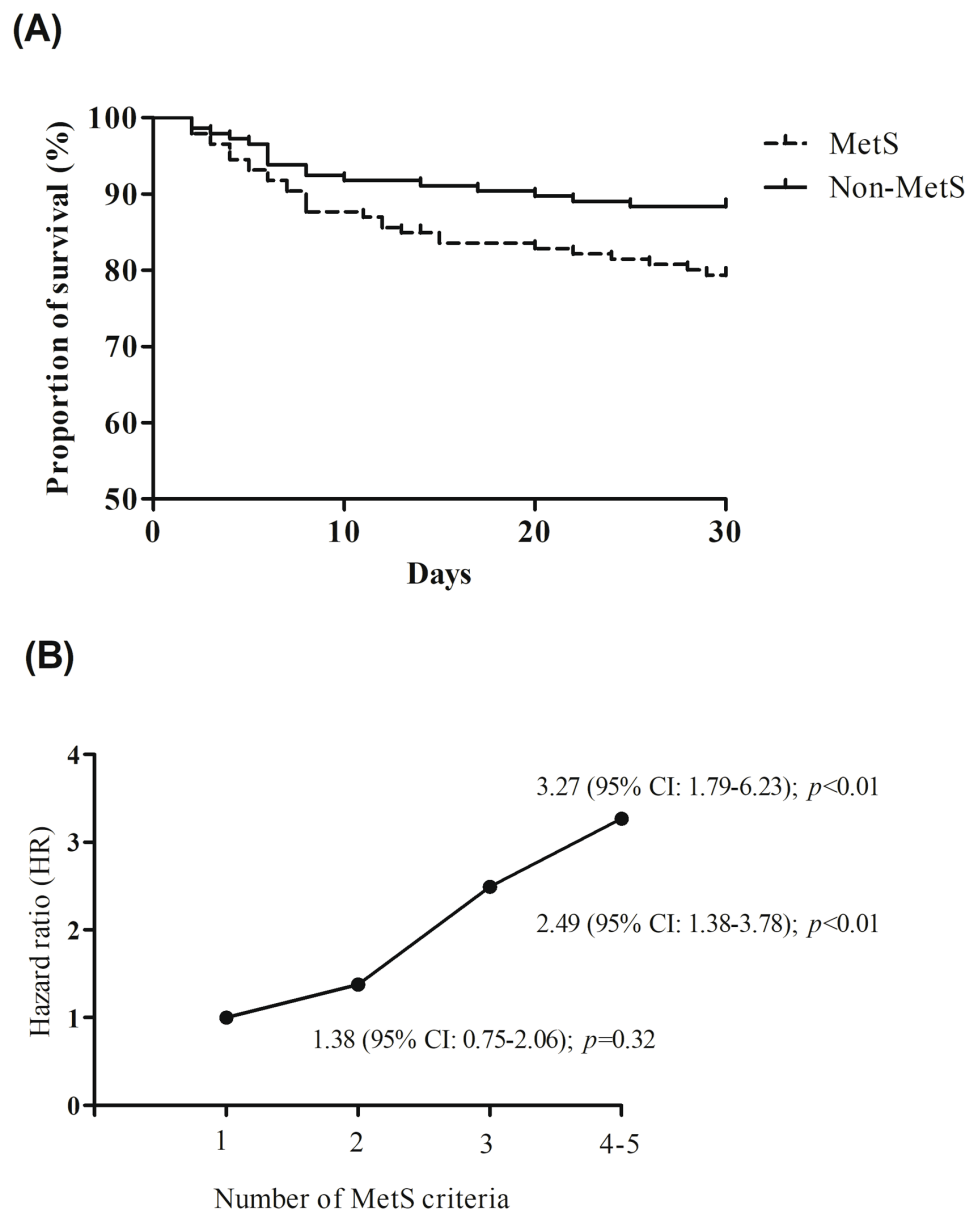
**(A)** Kaplan–Meier curves by the presence of metabolic syndrome (MetS) components. **(B)** The trend of effect-size estimates with the increasing number of MetS components.

As shown in Figure 2, the 3-month all-cause mortality rate was significantly higher in the MetS group compared to the non-MetS group (24.0% vs. 11.6%; *p* < 0.01). Kaplan–Meier survival analysis (Figure 3A) showed that patients with MetS had a significantly higher risk of 30-day all-cause mortality compared to those without (HR = 2.18, 95% CI: 1.22–3.89; *p* < 0.01). After adjusting for potential confounders, MetS remained independently associated with increased mortality (HR = 2.50, 95% CI: 1.35–4.60; *p* < 0.01) (Table 2). Additionally, a dose–response relationship was observed between the number of MetS components and the all-cause mortality (Figure 3B). Compared to the patients with 1 MetS component, those with 2, 3, and 4–5 components were associated with 1.38-fold (95% CI: 0.75–2.06; *p* = 0.32), 2.49-fold (95% CI: 1.38–3.78; *p* < 0.01), and 3.27-fold (95% CI: 1.79–6.23; *p* < 0.01) increased risks of all-cause mortality.

**Table 2:** Univariate and multivariate Cox and logistic regression analysis for primary and secondary outcomes.

Outcomes	Univariate analysis (MetS vs. Non-MetS)			Multivariate analysis (MetS vs. Non-MetS)		
	OR	95% CI	*p* value	OR	95% CI	*p* value
Primary outcome (Cox regression)						
Mortality at 3 months	2.18	1.22–3.89	<0.01	2.50	1.35–4.60	<0.01
Secondary outcomes (Logistic regression)						
mRS score of 0–2 at 3 months	0.46	0.29–0.74	<0.01	0.47	0.28–0.77	<0.01
SICH	2.10	1.09–4.05	0.03	2.40	1.17–4.92	0.02

For multivariate Cox or logistic regression analysis, potential confounding factors including age, sex, NIHSS score, TOAST classification, medical history, site of occlusion, ASPECTS and processing time were controlled. mRS, modified Rankin Scale; SICH, symptomatic intracranial hemorrhage.

### Secondary outcomes

Patients in the MetS group were less likely to achieve a good functional outcome (mRS score of 0–2) at 3 months compared to the non-MetS group (32.2% vs. 50.7%; *p* < 0.01). This association persisted after multivariate adjustment (adjusted OR = 0.47, 95% CI: 0.28–0.77; *p* < 0.01).

The incidence of SICH within 3 months was significantly higher in the MetS group compared to the non-MetS group (20.5% vs. 11.0%; *p* < 0.01). After adjusting for confounders, MetS remained an independent predictor of SICH (adjusted OR = 2.40, 95% CI: 1.17–4.92; *p* = 0.02).

## Discussion

In this prospective cohort study, we found that MetS was significantly associated with poor prognosis in patients with AIS after IVT. Specifically, patients with MetS exhibited higher all-cause mortality, lower rates of favorable functional outcomes (mRS 0–2), and an increased incidence of SICH at 3 months compared to those without MetS. These associations persisted after adjusting for potential confounders, suggesting that MetS is an independent predictor of adverse outcomes in this patient population.

The detrimental impact of MetS on stroke prognosis may be attributed to its multifaceted pathophysiological effects. Chronic low-grade inflammation and endothelial dysfunction, hallmarks of MetS, can impair neurovascular coupling and hinder neuronal repair processes (11, 12). Additionally, insulin resistance and hyperglycemia, key components of MetS, are known to increase oxidative stress, disrupt the blood–brain barrier, and impair cerebral autoregulation, which may exacerbate secondary brain injury after reperfusion therapies (13, 14). These mechanisms collectively contribute to worse neurological recovery, higher mortality, and increased hemorrhagic complications in patients with MetS.

Previous studies have reported inconsistent findings regarding the association between MetS and all-cause mortality. An observational study conducted by Suzuki et al. (15) indicated that MetS was significantly associated with increased mortality risk (HR = 1.26, 95% CI: 1.15–1.38). Similarly, the San Antonio Heart Study by Hunt et al. demonstrated a 1.47-fold (95% CI: 1.13–1.92) higher risk of all-cause mortality in individuals with MetS (16). In contrast, a study by Mi et al. (6) found no significant association between MetS and mortality. These discrepancies may be attributed to differences in cohort characteristics such as age, ethnicity, sex, treatment strategies, or other mortality risk factors. Our study strengthens the evidence linking MetS with increased mortality by using PSM to minimize selection bias and improve comparability between groups. Importantly, we identified a dose–response relationship between the number of MetS components and all-cause mortality risk, highlighting the cumulative impact of metabolic abnormalities on stroke outcomes. This finding underscores the importance of early identification and management of individual MetS components in AIS patients undergoing reperfusion therapies.

The reduced likelihood of favorable functional outcomes in patients with MetS underscores the need for early rehabilitation and tailored interventions to address metabolic disturbances in this population. In addition, our findings indicate a significantly increased risk of SICH among MetS patients receiving IVT. This result is consistent with previous studies suggesting that components of MetS—particularly hypertension, hyperglycemia, and dyslipidemia—may increase blood–brain barrier permeability and promote hemorrhagic transformation after thrombolysis (17, 18). For example, a study by Chen et al. reported a similar association between MetS and increased hemorrhagic complications following recanalization therapy for stroke (5). Conversely, other studies have found no significant relationship between MetS and SICH (19), possibly due to differences in patient characteristics, stroke subtypes, or baseline risk profiles. Taken together, our results contribute to the growing body of evidence emphasizing the importance for vigilant metabolic risk management—not only to improve functional recovery but also to reduce the incidence of serious complications such as SICH in AIS patients treated with IVT.

The strengths of our study include its prospective design, comprehensive data collection, and the application of PSM to control for baseline differences. However, several limitations should be noted. First, this was a single-center study, which may limit the generalizability of the findings. Second, although we adjusted for multiple confounding factors, residual confounding cannot be completely excluded. Third, we used the CDS criteria to define MetS, which may differ from other international definitions and affect comparisons with studies conducted in different regions. Future multicenter studies with larger cohorts and longer follow-up periods are warranted to validate our findings and explore targeted strategies to mitigate the adverse impact of MetS on stroke recovery.

In conclusion, MetS is independently associated with increased mortality, poor functional outcomes, and a higher risk of SICH in patients with AIS after IVT. These findings highlight the importance of comprehensive metabolic risk management to improve outcomes and reduce post-thrombolysis complications in this patient population.

## Data Availability

The raw data supporting the conclusions of this article will be made available by the authors, without undue reservation.
